# Dual-Target Electrochemical Sensor Based on 3D MoS_2_-rGO and Aptamer Functionalized Probes for Simultaneous Detection of Mycotoxins

**DOI:** 10.3389/fchem.2022.932954

**Published:** 2022-06-28

**Authors:** Yanyang Yu, Jie Han, Jiaqi Yin, Jingcheng Huang, Jing Liu, Lingjun Geng, Xia Sun, Wenping Zhao

**Affiliations:** ^1^ School of Agricultural Engineering and Food Science, Shandong University of Technology, Zibo, China; ^2^ Shandong Provincial Engineering Research Center of Vegetable Safety and Quality Traceability, Zibo, China; ^3^ Zibo City Key Laboratory of Agricultural Product Safety Traceability, Zibo, China

**Keywords:** dual-target, aptamer functionalized probes, MoS2-rGO, multiple mycotoxins, simultaneous detection

## Abstract

A dual-target aptamer functionalized probes (DTAFP) was applied for the detection of aflatoxin B1 (AFB1) and zearalenone (ZEN) simultaneously, which has not been reported. Meanwhile, two functional materials for signal amplification of the DTAFP were synthesized: 1) a three-dimensional molybdenum disulfide-reduced graphene oxide (MoS_2_-rGO) as a favorable loading interface; 2) a double-probes gold nanoparticles (AuNPs) modified by Thionin (Thi) and 6-(Ferrocenyl) hexanethiol (FC6S) as distinguishable and non-interfering signals. Mycotoxins on the electrode surface release into solution under the function of the DTAFP, leading a reduction of the differential peak impulse in signal response. Under the optimum conditions, the aptasensor exhibited a detection range of 1.0 pg mL^−1^–100 ng mL^−1^ for AFB1 and ZEN, with no observable cross reactivity. In addition, the aptasensor performed excellent stability, reproducibility, specificity, and favorable recovery in the detection of edible oil. This work demonstrated a novel method for the construction of a simple, rapid, and sensitive aptasensor in the detection of multiple mycotoxins simultaneously.

## Introduction

Mycotoxins are toxic secondary metabolites produced by filamentous fungi and have carcinogenic, mutagenic, teratogenic, neurotoxic, and immunotoxic. In general, mycotoxins contaminate crops and agricultural products during crop growth, harvest, storage and processin (Wang et al., 2017; Rushing and Selim, 2019). According to a conducted by the Food and Agriculture Organization of the United Nations (FAO), there are about 25% of the world’s crops were contaminated with mycotoxins, which caused huge economic losses and posed a serious threat to human health. Among the known mycotoxins, aflatoxin B1 (AFB1) has been classified as a Group 1 carcinogen by the International Agency for Research on Cancer (IARC) (Haque et al., 2020) and zearalenone (ZEN) has been classified as a Group 3B carcinogen (Al-Jaal et al., 2019). Unfortunately, these two mycotoxins commonly coexist in grains and have synergistic and additive toxicological effects on human and animal health, causing serious harm even at relatively low intakes (Wang et al., 2017; Peng et al., 2018). To meet the requirement of food safety, many countries have enacted regulations to monitor the problem of mycotoxin contamination, the EU (EC, No. 1126/2007) and China (GB2761-2017) set the maximum residue levels (MRLs) of 2 μg kg^−1^ and 5 μg kg^−1^ for AFB1 in cereals, respectively; 60 μg kg^−1^ and 200 μg kg^−1^ for ZEN in cereals, respectively. Six mycotoxins (including AFB1 and ZEN) must be detected and monitored in feed especially in China (GB2761-2017). Therefore, it is urgent to develop a simple and accurate method for highly sensitive and efficient detection of multiple mycotoxins (Deng et al., 2013) (Li et al., 2022).

In recent years, more and more modern analytical instrumental methods (i.e., high performance liquid chromatography (HPLC) (Hidalgo-Ruiz et al., 2019), liquid chromatography-tandem mass spectrometry (LC-MS) (Schlegel and Elsinghorst, 2020; Yang et al., 2020), ultra-high performance liquid chromatography-mass spectrometry (UHPLC-MS) (Manizan et al., 2018; Huang et al., 2022a) and etc.) have been used in the simultaneous detection of multiple mycotoxins. Although these methods have high sensitivity and reliable analytical results, they rely on sophisticated and expensive instruments, cumbersome sample pretreatment, long period detection and specialized personnel, which restrict their application for rapid detection in the field (Mahmoudi-Moghaddam et al., 2019). In comparison, electrochemical sensors with rapid response and simple operation as one of the promising alternatives due to its high sensitivity and portability, have drew a lot of attention of environmental pollution and food safety fields (Wang et al., 2019). Aptamer as a new recognition element was obtained from the “Systematic Evolution of Ligands by Exponential Enrichment (SELEX).” Compared with traditional antibody, aptamer possess many competitive advantages for sensing applications, including excellent stability during long-term storage, low-cost chemical synthesis, versatility in modification, low toxicity and lack of immunogenicity (Li et al., 2021) (Liu et al., 2022). Up to now, many specific aptamers combined with surface enhanced raman ecattering (SERS) (Li et al., 2017), fluorescence (Yang et al., 2017), colorimetric (Setlem et al., 2020), surface plasmon resonance (SPR) (Mahmoudpour et al., 2019), chemiluminescent (ECL) (Yao et al., 2019) and electrochemical (Qian et al., 2020) techniques have been well developed in the detection of mycotoxins. In addition, aptamer still remains its outstanding bind-to-target performance even after repeated denaturalization and renaturation. Therefore, aptamer was a more suitable tool in complex environments where multiple targets were detected simultaneously (Yue et al., 2014) (Shekari et al., 2021).

In order to obtain an ideal electrochemical sensor, it was necessary to select a suitable substrate material to modify the electrode (Wang et al., 2016). Molybdenum disulfide (MoS_2_) nanomaterials have attracted a lot of attention because of the large specific surface area, low toxicity and embeddable morphology (Shen et al., 2019; Zhang et al., 2020). Furthermore, MoS_2_ nanosheets have been shown to spontaneously adsorb single-stranded DNA (ssDNA) through van der Waals force between the nucleobases and the base surface of the nanosheets (Zhu et al., 2013), so it can adsorb aptamers spontaneously. However, a previous report shown that MoS_2_ nanosheets present poor electrochemical sensing properties and agglomeration due to its inherent semiconductor properties (Wang et al., 2018). As a typical two-dimensional material, reduced graphene oxide (rGO) and reduced graphene-based composites had been widely used as substrate material for aptasensor due to their large specific surface area, good electrical conductivity and anti-agglomeration behavior (Lyu et al., 2021), such as DNA (Malmir et al., 2021), protein (Sonuc Karaboga and Sezginturk, 2020) and biomarker (Kasturi et al., 2021) detections. Therefore, the composite material coupling MoS_2_ with rGO can be an excellent substrate material for electrochemical sensing (Han et al., 2021). To further improve the connection between biomolecules and the substrate material, gold nanoparticles (AuNPs) can be attached to MoS_2_-rGO nanocomposite, the combination of both can further enhance the electrochemical properties and achieve signal amplification.

Based on the information above, a dual-target electrochemical sensor based on 3D MoS_2_-rGO and aptamer functionalized probes were constructed for the simultaneous detection of AFB1 and ZEN. The present work was followed: 1) Thionin (Thi) and 6-(Ferrocenyl) hexanethiol (FC6S) as redox probes, shown good response signals near the potentials of −0.2 and 0.45 V, respectively, with a relatively wide potential window (>0.6 V) to avoid mutual interference of the two signalsthe; 2) the high affinity of AuNPs for sulfhydryl molecules was used to immobilize sulfhydrylated aptamers and dual probes; 3) 3D MoS_2_-rGO enhanced the immobilization amount of the DTAFP and facilitated the electrons transfer between the probes and the electrode surface. In the present of mycotoxins, the DTAFP bound with their corresponding mycotoxins to form complexes and fell off the electrode surface, reducing the electroactive substances on the electrode surface and lowering the corresponding current signals. Therefore, the simultaneous detection of AFB1 and ZEN can be achieved by analyzing the current changes before and after the addition of mycotoxins.

## Materials and Methods

### Materials

Gold (III) chloride trihydrate (HAuCl_4_⋅3H_2_O), 6-(Ferrocenyl) hexanethiol (FC6S), Thionin (Thi), 6-mercapto-1-hexanol (MCH), Sodium molybdate dihydrate (Na_2_MoO_4_-2H_2_O), aflatoxin B1 (AFB1), and zearalenone (ZEN) were purchased from MACKLIN Biochemical Co., Ltd. (Shanghai, China). Deoxynivalenol (DON), T-2, aflatoxin G1 (AFG1), aflatoxin M1 (AFM1), and Ochratoxin A (OTA) were purchased from Alta Technology Co., Ltd. (Tianjin, China). The aptamer sequences were selected based on previously reported literature (Yang Y. et al., 2021; Zhu et al., 2021), which were synthesized by Sangon Biotech Co., Ltd. (Shanghai, China). AFB1 aptamer:

5′-SH-GTTGGGCACGTGTTGTCTCTCTGTGTCTCGTGCCCTTCGCTAGGCCCACA-3′

ZEN aptamer:

5′-SH-TCATCTATCTATGGTACATTACTATCTGTAATGTGATATG-3′

1×TE Buffer and trimethylaminomethane hydrochloride buffer (Tris-HCl) were obtained from Sangon Biotech Co., Ltd. (Shanghai, China). Monolayer graphene oxide (GO) powder was purchased from XFNANO Materials Tech Co., Ltd. (Nanjing, China). Thiourea (CH_4_N_2_S) and trisodium citrate (C_6_H_5_Na_3_O_7_) were purchased from Sinopharm chemical reagent Co., Ltd. (Shanghai, China). The oil samples were obtained from the local supermarket (Zibo, Shandong, China). The remaining chemical reagents used were commercially available in analytical purity, and all solutions were prepared with ultrapure water.

### Instrumentation

The electrochemical measurements were performed on a CHI660D electrochemical workstation (Chenhua Instruments Co., Ltd. China). A three-electrode system was used for this experiment: a gold electrode (AuE) as the working electrode, a saturated Ag/AgCl electrode as the reference electrode, and a platinum electrode (Pt) as the counter electrode. Ultrapure water (18.2 MΩ) was obtained from Barnstead GenPure system (Thermo Fisher Scientific, Waltham, MA). UV-Vis spectra was obtained by Varioskan LUX (Heros Technology Co., Ltd. China) and the morphology of the samples was characterized by scanning electron microscopy (SEM, FEI Sirion 200, United States).

### Preparation of MoS_2_-rGO Nanocomposite

The MoS_2_-rGO was synthesized according to the previous literatures (Wang Y. et al., 2020; Yin et al., 2021). 30 mg of GO and 0.4 g of Na_2_MoO_4_-2H_2_O were dispersed into 30 mL of ultrapure water, and sonicated for 30 min to obtain a homogeneous mixture. Then 0.63 g thiourea was added to the mixture and sonicated for 30 min. The mixture was transferred to a 50 mL Teflon-lined stainless steel autoclave and heated at 180°C for 12 h. After cooling to room temperature naturally, the mixture was repeatedly centrifuged and washed three times with ethanol and ultrapure water at 13,000 rpm min^−1^ for 10 min to remove the unreacted portion. Finally, the obtained mixture was dried at 65°C, dispersed in ultrapure water (1 mg mL^−1^) and stored for further use.

### Preparation of Aptamer Functionalized Probes

AuNPs was prepared according to the previous experimental method (Yan et al., 2012). 100 mL of 0.01% HAuCl_4_⋅3H_2_O was heated until boiling, then 2.5 mL of 1% trisodium citrate added to the above solution. The mixture was kept stirring and heating for 10 min until the solution changed from gray to stable burgundy. After the solution was cooled naturally to room temperature with stirring, washed it with ultrapure water and restored to the original volume.

The 1 μM AFB1 aptamer (DNA1) solution and AuNPs were mixed and homogenized for 8 h. The mixture was then centrifuged at 7000 rpm min^−1^ for 10 min to remove the supernatant, and redispersed into 1×TE buffer. After that, 10 mg mL^−1^ of Thi was added and mixed for 8 h. The above solution was centrifuged to remove the supernatant and restored the original volume to form DNA1-AuNPS-Thi. Similarly, 1 μM of ZEN aptamer (DNA2) solution and 20 mM of FC6S were coupled to AuNPs in the same way to form DNA2-AuNPs-FC6S. The volume ratios of Thi: AuNPs: DNA1 and FC6S: AuNPs: DNA2 were 1:6:6 and 2:3:3, respectively.

### Fabrication of the Aptasensor

Aluminum powders with particle sizes of 1 μm, 0.3 and 0.05 μm were used to polish AuE on suede to obtain a mirror-like surface, and which was cleaned with ultrapure water. Then, the electrode was sonicated in ultrapure water and anhydrous ethanol for 60 s, and dried with nitrogen gas. The pretreatment effect of electrode was in 5 mM [Fe(CN)_6_]^3-/4-^ solution (pH 7.5, containing 0.1 M KCl). The pretreated electrode was used for further modification with the peak potential difference of the cyclic voltammogram (CV) curve less than 100 mV.

The fabrication steps of the aptasensor were as follows: 5 μL of MoS_2_-rGO was added on the electrode surface and incubated for 2 h at 25°C to immobilize MoS_2_-rGO on the electrode surface through Au-S bonds. Subsequently, 5 μL of DNA1-AuNPs-Thi and 5 μL of DNA2-AuNPs-FC6S were added dropwise to the MoS_2_-rGO/AuE surface and incubated at 25°C for 2 h. After that, 5 μL of MCH was added dropwise to the modified electrode surface to block the nonspecific binding site. The obtain electrode was then rinsed with ultrapure water and dried with nitrogen gas. Finally, the prepared aptasensor were stored in a dry environment at 4°C.

### Electrochemical Detection

Cyclic voltammogram (CV) was performed in the solution containing 5 mM [Fe(CN)_6_]^3-/4-^ (pH 7.5, containing 0.1 M KCl) with potential window of −0.1 and 0.6 V and a scan rate of 100 mV s^−1^. Electrochemical impedance spectroscopy (EIS) was performed in the same solution with an amplitude of 5 mV and a frequency range of 0.1 Hz–10 kHz. Zview software was used to fit Nyquist plots based on the Randles equivalent circuit. Differential pulse voltammetry (DPV) was performed in 50 mM Tris-HCl solution with a scan range of −0.5–0.7 V and a pulse amplitude of 50 mV, 0.05 s pulse width and 0.5 s pulse period. All measurements were performed at room temperature of 25°C.

### Preparation of Real Samples

The real samples were prepared according to the previous report (Jiang Y.-Y. et al., 2021) 5 g of the uncontaminated oil and 1 g of sodium chloride were added into 25 mL of methanol extract (V methanol: V ultrapure water = 80:20), and then the mixture was shaken completely for 30 min at 25°C on a horizontal shaker and then centrifuged at 6500 rpm min^−1^ for 10 min to obtain the 15 mL of the upper extract. After that, the obtained upper extract was filtered twice through a 0.22 μm glass fiber filter paper membrane, and 30 mL of PBS solution (pH 7.4, 0.1 M) was added into it. Finally, this filtrate was was prepared with different concentrations of AFB1 and ZEN for real samples experiments.

## Results

### Principle of Simultaneous Detection of AFB1 and ZEN

**FIGURE 1:**
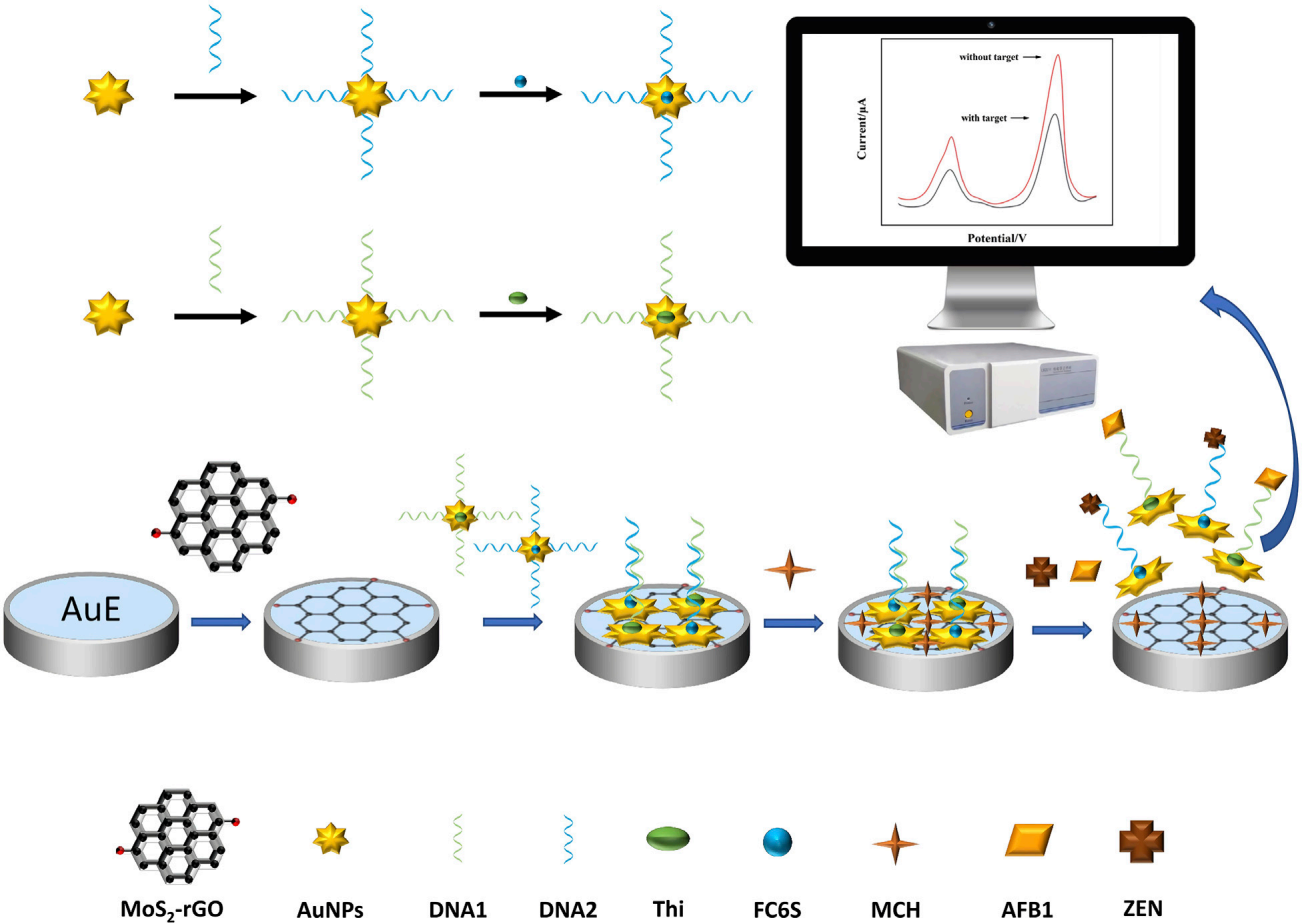
Schematic diagram of dual-target electrochemical sensor for multiplexed detection of mycotoxins.

The principle for simultaneous detection of AFB1 and ZEN based on MoS_2_-rGO and aptamer functionalized probes was shown in Figure 1. The MoS_2_-rGO and AuNPs could amplify the dual current signals of the probes Thi and FC6S without the target. In the present of mycotoxins, two aptamer functionalized probes bound with their corresponding mycotoxins to form complexes DNA1-AuNPS-Thi/AFB1 and DNA2-AuNPS-FC6S/ZEN, and fell off the electrode surface, the electroactive substances (Thi and FC6S) on the electrode surface were reduced, and accordingly the two characteristic peaks caused by the two electroactive substances were also reduced. Therefore, the simultaneous detection of AFB1 and ZEN can be achieved by analyzing the current changes before and after the addition of mycotoxins.

### Characterization of Composite

**FIGURE 2:**
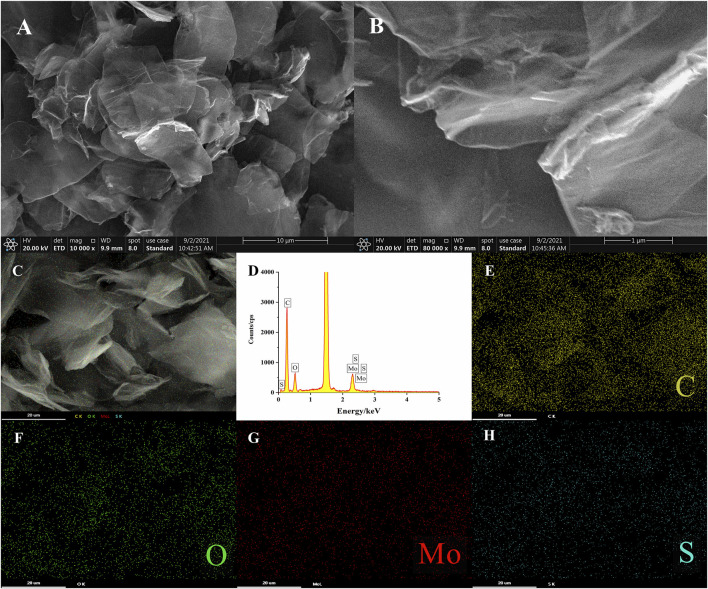
**(A,B)** SEM images of MoS_2_-rGO; **(C–H)** EDS mapping analysis of MoS_2_-rGO.

The morphologies of MoS_2_-rGO composite were characterized by SEM. It could be observed from Figure 2A that the whole composite nanomaterial shown a peony flower-like structure, in which the graphene oxide has been reduced and shown a cicada wing shape with a folded structure. Meanwhile, there were lots of folds on the surface of rGO, which were due to the coating of MoS_2_ nanosheets (Figure 2B). A randomly selected area (Figure 2C) was analyzed by Energy Dispersive Spectroscopy (EDS) and the composite was found to contain four elements (Figure 2D), It can be seen that the C (59%) (Figure 2E), O (16%) (Figure 2F), Mo (11%) (Figure 2G), S (14%) (Figure 2H), and elements in the MoS_2_-rGO composite were uniformly distributed in the material without serious aggregation and other impurity elements. On the one hand, the interaction between rGO and MoS_2_ nanosheets promoted the electrons transfer between the active MoS_2_-rGO and electrolyte interfaces, which increased the electrical conductivity of the composite (Chen et al., 2021). On the other hand, rGO not only provided a support structure for the growth of MoS_2_, but also formed a large number of pores inside it, these pores provided sufficient sites for the reaction of aptamer functionalized probes on the composite (Yang H. et al., 2021). The above results indicated that MoS_2_-rGO nanocomposites had been successfully synthesized.

### Characterization of the Aptamer Functionalized Probes

**FIGURE 3:**
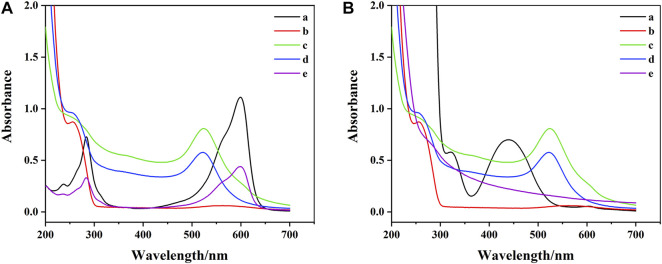
**(A)** UV-vis spectrum of aptamer functionalized probes: (a) Thi, (b) DNA1, (c) AuNPs, (d) DNA1-AuNPs, (e) DNA1-AuNPs-Thi. **(B)** UV-vis spectrum of aptamer functionalized probes: (a) FC6S, (b) DNA2, (c) AuNPs, (d) DNA2-AuNPs, (e) DNA2-AuNPs-FC6S.

To further explore the construction process of DTAFP, Ultraviolet-visible (UV-Vis) absorption spectra was used to characterize DNA1-AuNPs-Thi and DNA2-AuNPs-FC6S. As shown in Figure 3, DNA1 and DNA2 shown a characteristic peak at 256 nm (curve b). The characteristic peak at 524 nm was characteristic of the plasma band on the surface of gold nanoparticles (curve c) (Guo et al., 2021) and after adding DNA1 and DNA2 to AuNPs, the absorption peaks appeared at 254 and 522 nm (curve d), which demonstrated that the DNA1 and DNA2 had been immobilized on AuNPs (Devi et al., 2019). In Figure 3A, the Thi shown two characteristic peaks at approximately 284 and 600 nm (curve a), which were attributed to the π-π* leap of the aromatic ring and the n-π* leap of the C=N bond, respectively (Zhou et al., 2016). After binding to DNA1-AuNPs, the characteristic peaks of Thi at 600 and 284 nm were shifted to 599 and 283 nm (curve e) due to the interaction between the NH_2_-terminated in Thi and AuNPs (Han et al., 2020). In Figure 3B, the FC6S shown two characteristic peaks at approximately 440 and 320 nm (curve a). After modification by DNA2-AuNPs, the characteristic peak of FC6S disappeared completely (curve e) due to the cleavage of the S-H bond and the formation of a new bond, the Au-S bond. In conclusion, these results clearly indicated that the desired DNA1-AuNPs-Thi and DNA2-AuNPs-FC6S aptamer functionalized probes had been successfully prepared.

### Electrochemical Behavior of the Aptasensor

**FIGURE 4:**
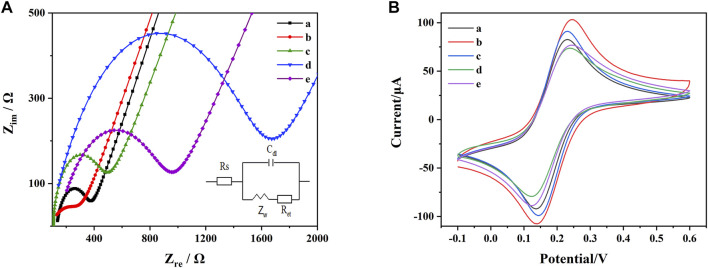
**(A)** EIS of the AuE with different modifications: (a) AuE, (b) AuE/MoS_2_-rGO, (c) AuE/MoS_2_-rGO/DNA1-AuNPs-Thi and DNA2-AuNPs-FC6S, (d) AuE/MoS_2_-rGO/DNA1-AuNPs-Thi and DNA2-AuNPs-FC6S/MCH, (e) AuE/MoS_2_-rGO/DNA1-AuNPs-Thi and DNA2-AuNPs-FC6S/MCH/AFB1 and ZEN. **(B)** CV of the AuE with different modifications: (a) AuE, (b) AuE/MoS_2_-rGO, (c) AuE/MoS_2_-rGO/DNA1-AuNPs-Thi and DNA2-AuNPs-FC6S, (d) AuE/MoS_2_-rGO/DNA1-AuNPs-Thi and DNA2-AuNPs-FC6S/MCH, (e) AuE/MoS_2_-rGO/DNA1-AuNPs-Thi and DNA2-AuNPs-FC6S/MCH/AFB1 and ZEN.

Each step of the electrode modification was carefully examined by EIS. The experimental results were fitted according to the Randles equivalent circuit. The fitted parameters included electrolyte solution resistance (Rs), constant phase element (Q), electrons transfer resistance (R_et_) and Warburg impedance (Zw). Among these parameters, Rs is the resistance of the electrolyte solution; Q is the double-layer capacitance of the system and Zw is the diffusion resistance of the system. R_et_ reflected the electrons transfer kinetic occurring at the electrode surface and used to research the structure and recognition process of the aptasensor. As shown in Figure 4A, the R_et_ value at AuE was estimated to be around 400 Ω (curve a), which decreased to around 250 Ω after the assembly of MoS_2_-rGO due to its high electrons transfer rate (curve b). Subsequent to the assembly of the aptamer functionalized probes, electrons transfer was significantly blocked, thus leading to the increase of R_et_ (around 500 Ω) (curve c). After adding MCH to block the non-specific binding site, the R_et_ further increased to around 1700 Ω (curve d). Finally, after the addition of AFB1 and ZEN, the DNA1-AuNPs-Thi/AFB1 and DNA2-AuNPs-FC6S/ZEN complexes were formed, leading to the breakage of DNA1-AuNPs-Thi and DNA2-AuNPs-FC6S from the electrode and decreased in R_et_ to around 950 Ω (curve e).

[Fe(CN)_6_]^3-/4-^ was used as a redox probe to observe the electron transfer of microstructural changes at the electrode surface. As shown in Figure 4B. The bare AuE exhibited a significant reversible redox behavior due to the high level of electrons transfer between the [Fe(CN)_6_]^3-/4-^ solution and the electrode surface (curve a). Modification with MoS_2_-rGO increased the electrical conductivity of the nanocomposite, which led to a significant increase in the redox peak currents (curve b). When DNA1-AuNPs-Thi and DNA2-AuNPs-FC6S were attached to AuE/MoS_2_-rGO, a decrease in the redox peak currents of [Fe(CN)_6_]^3-/4-^ were observed, probably due to the lower conductivity of the aptamer (curve c). When the MCH was added, the redox peak currents were reduced to a minimum (curve d). After further incubation with AFB1 and ZEN, the redox peak currents were further enhanced (curve e). The results of EIS characterizations and CV characterizations followed the same trend. All these experiments indicated that the aptasensor has been successfully prepared as expected for the simultaneous determination of AFB1 and ZEN.

### Optimization of Experimental Conditions

In order to maximize the detection performance of the aptasensor, three key parameters including the concentration of the aptamer, incubation time and pH value were optimized in this work.

**FIGURE 5:**
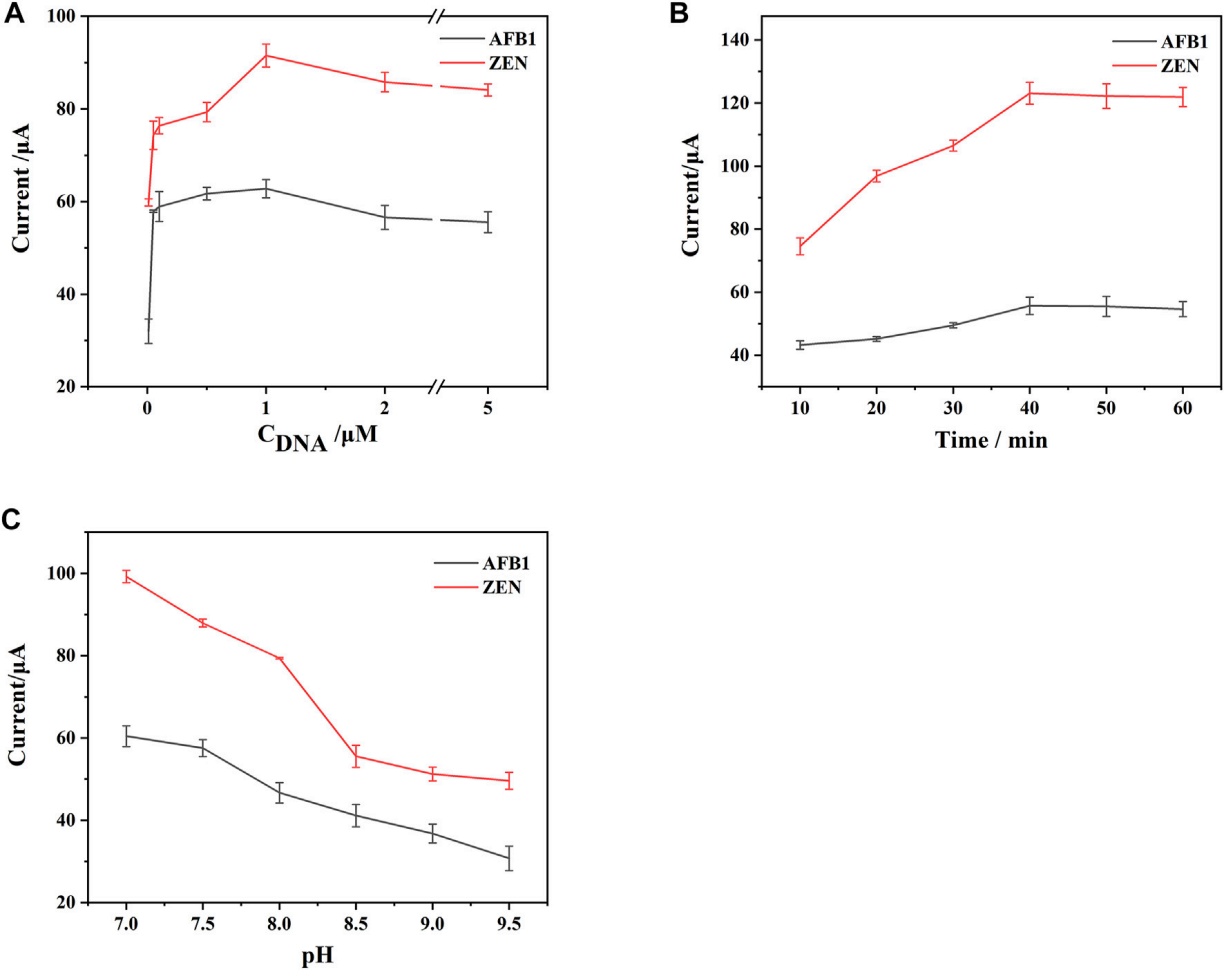
Effects of the key parameters on the performance of the constructed aptasensor: **(A)** concentration of DNA. **(B)** incubation time. **(C)** pH value. Error bars represent the standard deviations of three independent measurements.

As shown in Figure 5A, the DPV response signals were recorded at different concentrations of the aptamer, the DPV response signals of both Thi and FC6S increased significantly as the aptamer concentration increased from 0.01 to 1 μM, and then gradually decreased as the concentration range decreased from 1 to 5 μM for both. It indicated that when the concentration of the aptamer was 1 μM, the aptamer had reached the saturation state on the electrode surface, and excessive concentration of the aptamer would form stacking and entanglement on the electrode surface, which would affect the binding of aptamer with target (Li et al., 2018). Therefore, the concentration of aptamer was chosen 1 μM to prepared for DNA1-AuNPS-Thi and DNA2-AuNPS-FC6S aptamer functionalized probes.

The incubation time for aptamer-target binding was also explored in Figure 5B. Initially, the DPV response signal increased with the incubation time because more target binding specifically to aptamer. Then the DPV response signal leveled off with time prolonging, probably because the binding of the target to the aptamer gradually reached the saturation state. For the purpose of rapid detection, 40 min was chosen as the optimal incubation time.

A recent study found that Tri-HCl buffer takes an active role in DNA cleavage (Huang et al., 2022b), so Tri-HCl buffer was selected to provide a positive reaction environment for DTAFP. Since Tris-HCl was a neutral and weakly basic buffer, the prepared aptasensor were tested in Tris-HCl buffer ranging from pH 7 to pH 9.5, Figure 5C observed that the DPV response signal reached a maximum at pH 7. Therefore, the buffer at pH 7 was chosen as the test solution in further experiment.

### Cross-Reactivity of the Aptasensor

**FIGURE 6:**
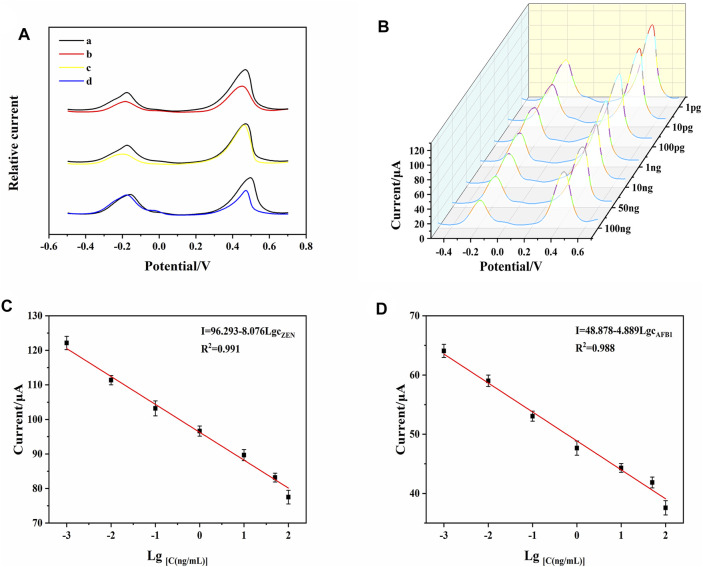
**(A)** DPV signals of AFB1 and ZEN for investigation of cross-reactivity. **(B)** DPV responses of the developed aptasensor toward AFB1 and ZEN with different concentrations. **(C)** calibration curve of AFB1. **(D)** calibration curve of ZEN. Error bars represent the standard deviations of three independent measurements.

In the context of simultaneous detection of multiple analytes, it is crucial to exclude cross-reactivity between analytes. To further evaluate the cross-reactivity of the DTAFP-based sensors, DPV responses were tested for AFB1, ZEN, and their mixtures, respectively. As shown in Figure 6A, in the absence of target mycotoxins, the aptasensor has two DPV response signals at around −0.2 V (Thi) and 0.45 V (FC6S) (curve a), and both peak currents decreased simultaneously when AFB1 was added in response with ZEN (curve b). When AFB1 response was added to the aptasensor, the response signal of Thi decreased significantly, while the response signal of FC6S remained essentially unchanged (curve c). When ZEN was added alone, the response signal of Thi basically maintained its original response, while the response signal of FC6S decreased significantly (curve d). Therefore, the designed aptasensor perform the detection of AFB1 and ZEN simultaneously without cross-reactivity.

### Analytical Performance of the Aptasensor

The sensing performance corresponding to different concentration of AFB1 and ZEN was tested under the optimal conditions. As shown in Figure 6B, when the aptasensor was exposed to mycotoxins, DNA1-AuNPS-Thi/AFB1, and DNA2-AuNPS-FC6S/ZEN were released from the electrode surface, resulting in a decrease of the peak currents. As the concentration of mycotoxins increased, the current of Thi and FC6S also decreased at the same time. To quantitatively investigate the analytical properties, calibration curves were plotted with the target mycotoxins concentration and DPV response signal as the horizontal and vertical coordinates, respectively. Figures 6C,D shown a linear correlation between the DPV response signals of Thi and FC6S with AFB1 and ZEN between 1 pg mL^−1^–100 ng mL^−1^. For the analysis of AFB1, the regression equation was determined as I (μA) = 48.878–4.889 LgcAFB1 (R^2^ = 0.988) and limit of detection (LOD) was 0.0003 ng mL^−1^; for the analysis of ZEN, the regression equation was I (μA) = 96.293–8.076 LgcZEN (R^2^ = 0.991) and LOD was 0.0003 ng mL^−1^. These results indicated that the present strategy is capable to determine AFB1 and ZEN simultaneously, showing excellent sensitivity, detection limit and linear range. Table 1 shown the comparison of the present aptasensor with other reported methods for detection of AFB1 and ZEN and the results shown that the aptasensor has a wide dynamic range and low detection limit.

**TABLE 1:** Analytical performances of various reported methods for AFB1 and ZEN detection.

Method	Linear range (ng mL^-1^)		LOD (ng mL^−1^)		References
	AFB1	ZEN	AFB1	ZEN	
HPLC-MS	0.1–100	0.1–100	0.025-	0.025	Al-Taher et al., (2013)
SPR	0.99–21.92	10.37–103.31	0.59	7.07	Wei et al., (2019)
Colorimetry	0.05–10	-	0.03	–	Jiang et al., (2021a)
Fluorescence	0.015–0.5	0.25–2.5	0.0093	0.102	Li et al., (2022)
Chemiluminescence	0.5–40	–	0.2	–	Sun and Zhao, (2018)
Electrochemical SWV	0.63–156.3	–	0.63	–	Wang et al., (2020a)
Electrochemical ACV	0.01–3.0	–	0.0043	–	Zhu et al., (2021)
Electrochemical DPV	0.001–100	0.001–100	0.0003	0.0003	This work

### Specificity, Reproducibility and Stability of the Aptasensor

**FIGURE 7:**
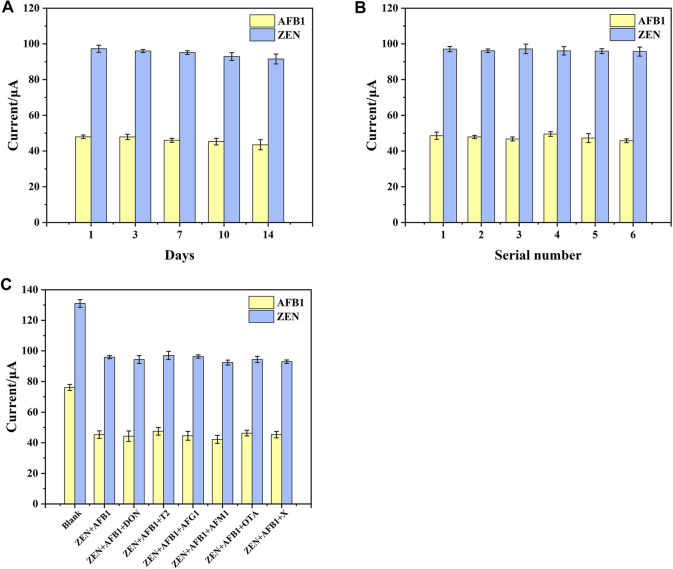
**(A)** Stability of aptasensor. **(B)** Reproducibility of aptasensor. **(C)** The interference research of aptasensor toward different compounds. X represents ZEN + AFB1+DON + T2+AFG1+AFM1+OTA. Error bars represent the standard deviations of three independent measurements.

Fifteen electrodes were prepared under the same conditions, and individual electrodes were stored in a refrigerator at 4°C. As shown in Figure 7A, the DPV response signals of the electrodes to AFB1 and ZEN after 7 days were 95.94% and 97.8% of the initial (RSD 2.43% and 1.08%, respectively). The DPV response signals of the electrodes to AFB1 and ZEN after 14 days were 90.69% and 94.11% of the initial (RSD 6.44% and 3.04%, respectively). The results indicated that the aptasensor performed good stability, which could be attributed to the self-assembly of MoS_2_-rGO and DTAFP on the AuE surface by Au-S bonding layer by layer.

Reproducibility was also one of the important criteria to evaluate a sensor. To verify the reproducibility of the aptasensor, 6 batches of 3 independent electrodes were prepared under the same conditions to detect AFB1 and ZEN. As shown in Figure 7B, The RSD values of the electrode were 3.09% and 1.98%, suggested that the aptasensor had good reproducibility.

According to the previous reports, a number of mycotoxins including DON, T2, AFG1, AFM1, and OTA frequently coexist with AFB1 and ZEN in cereals (Gruber-Dorninger et al., 2017; Rai et al., 2020). Here, they were served as interfering substances to research the selectivity of the aptasensor. As shown in Figure 7C, the differences in DPV response signals in the presence of interferents were very small for all interfering substances at a concentration of 5 ng mL^−1^ and all target substances at the concentration of 1 ng mL^−1^. These subtle signal changes suggested that interfering substances had little effect on the aptasensor. This may be attributed to the high affinity of the aptamer for AFB1 and ZEN molecules, thus enabling the simultaneous determination of ZEN and FB1 from complex sample matrices without purification and enrichment.

### Real-Life Sample Analysis

To verify the applicability of the method, recovery experiments were carried out in real samples to evaluate the accuracy of the designed aptasensor. A series of fixed concentrations of AFB1 and ZEN were added to corn oil and peanut oil. The samples were tested three times (*n* = 3) and the results were shown in Table 2, the recoveries are ranging from 96.61% to 109.14% for AFB1 and 90.16%–110.4% for ZEN, with relative standard deviations (RSD) less than 10%. At the same time, the recoveries of both mycotoxins were analyzed by the classical LC–MS/MS method, which ranged from 78.37% to 108.7% for AFB1 and 102.23%–108.15% for ZEN (Table 3), in agreement with that of the established method. In addition, the results from different sample matrices may influence the analysis results. After testing in high concentrations of mycotoxins by the established method, it was found that the effect of the oil sample matrix was minimal (Table 2). While the reliability of the conclusion was checked with classical LC–MS/MS method (Table 3). The results shown that the established method can be used for multiplex mycotoxin screening analysis in real samples.

**TABLE 2:** AFB1 and ZEN recoveries in samples analyzed by the proposed sensing platform (n = 3).

Analytes	Added (ng mL^−1^)		Found (ng mL^−1^)		Recovery (%)		RSD (%)	
	AFB1	ZEN	AFB1	ZEN	AFB1	ZEN	AFB1	ZEN
corn oil	10	10	9.66	9.02	96.61	90.16	1.8	5.54
	20	20	21.83	22.08	109.14	110.4	1.2	3.43
	100	100	99.95	104.71	99.95	104.71	2.97	3.77
peanut oil	10	10	10.19	10.97	101.86	109.65	2.39	1.99
	20	20	21.53	21.98	107.64	109.9	2.35	3.7
	100	100	105.93	105.93	105.93	105.93	6.56	7.03

**TABLE 3:** AFB1 and ZEN recoveries in samples analyzed by the LC–MS/MS.

Analytes	Added (ng mL^−1^)		Found (ng mL^−1^)		Recovery (%)		RSD (%)	
	AFB1	ZEN	AFB1	ZEN	AFB1	ZEN	AFB1	ZEN
corn oil	10	10	9.74	10.5	97.4	104.95	3.92	6.4
	20	20	17.31	20.45	86.53	102.23	2.17	4.46
	100	100	78.37	102.83	78.37	102.83	0.08	0.52
peanut oil	10	10	10.87	10.82	108.7	108.15	0.26	3.73
	20	20	18.36	20.81	91.78	104.03	2.74	6.9
	100	100	83.23	104.46	83.23	104.46	3.85	1.06

## Conclusion

In summary, MoS_2_-rGO, and DTAFP composites were prepared in this work, and an electrochemical sensing platform for the simultaneous detection of multiple mycotoxins was constructed based on these composites. Due to the unique structural characteristics of the sensing platform with self-assembled layers and the electrochemical properties with double signal amplification, it shown high sensitivity, wide linear range, low detection limit, good anti-interference, reproducibility and stability for simultaneous detection of AFB1 and ZEN. The usefulness of the aptasensor was verified by detecting AFB1 and ZEN in peanut oil and corn oil, and the results were satisfactory. Moreover, compared with previously reported strategies, the aptasensor can omit the complementary strand as an immobilized nucleic acid aptamer carrier, which not only saves experimental materials and reduces experimental costs, but also reduces the preparation time of the aptasensor. Finally, due to its simple material synthesis and low detection cost, it could replace many time-consuming and expensive detection devices and detection methods to ensure food safety and human wellness. Our design concept of the sensor can also be easily extended to detect a large number of other substances simultaneously by using different probes when specific aptamers are available.

## Data Availability

The raw data supporting the conclusions of this article will be made available by the authors, without undue reservation.
